# A 21-Year-Old Female with Invasive Breast Cancer within a Benign Phyllodes Tumor

**DOI:** 10.70352/scrj.cr.25-0503

**Published:** 2026-01-21

**Authors:** Hisami Yoneda, Yoshiko Shimizu, Yuan Bae, Tomo Osako, Akiko Ogiya

**Affiliations:** 1 Department of Breast Surgery, Japanese Red Cross Medical Center, Tokyo, Japan; 2 Department of Pathology, Japanese Red Cross Medical Center, Tokyo, Japan; 3 Division of Pathology, Cancer Institute, Japanese Foundation for Cancer Research, Tokyo, Japan; 4 Department of Pathology, The Cancer Institute Hospital of Japanese Foundation of Cancer Research, Tokyo, Japan

**Keywords:** phyllodes tumor, breast cancer, invasive ductal carcinoma, young

## Abstract

**INTRODUCTION:**

Although breast cancer occurring within a phyllodes tumor (PT) has been reported, it is extremely rare in young patients. Herein, we describe a case of breast cancer complicated by a PT in a 21-year-old female.

**CASE PRESENTATION:**

A 21-year-old female was referred to our hospital with a rapidly growing breast mass and suspected PT. Mammography revealed a well-defined, high-density mass. Ultrasonography revealed a blood-flow-rich hypoechoic mass with multiple slit structures, and MRI revealed a heterogeneous high signal on T2 weighted image with some diffusion restriction. A core needle biopsy revealed fibroepithelial lesions, and the lack of stromal changes suggested a high possibility of fibroadenoma. Based on the clinical and imaging findings, we considered the possibility of the PT being more than borderline malignant and planned tumor resection with a safety margin. The postoperative pathology revealed a benign PT complicated by invasive ductal carcinoma, with a predominance of intraductal carcinoma.

**CONCLUSIONS:**

We report the case of a 21-year-old female with breast cancer occurring within a PT. The presence of cancer was difficult to predict preoperatively based on the patient’s young age and imaging findings.

## Abbreviations

ADC
: apparent diffusion coefficient
DCIS
: ductal carcinoma *in situ*
FA
: fibroadenoma
HBOC
: hereditary breast and ovarian cancer
IDC
: invasive ductal carcinoma
PT
: phyllodes tumor
RT
: radiation therapy
SN
: sentinel lymph node biopsy
T2WI
: T2 weighted image
US
: ultrasonography

## INTRODUCTION

Breast cancer is a malignant tumor derived from ductal epithelial cells and has a different age predilection in Caucasians and Asians. The incidence rate increases with age in Caucasians but begins at a younger age in Asians than in Caucasians. In addition, the incidence peaks among Asians in their 40s to 70s and declines thereafter. In Japan, the incidence of breast cancer begins in individuals in their 20s, but the rate is very low, accounting for <10% of all breast cancers.^1)^

PT are unique to the mammary gland and contain a mixture of ductal epithelial cells and underlying connective tissue. They account for <1% of all breast tumors and are more common in patients in their late 40s.^2)^ They are classified as benign, borderline malignant, or malignant, according to their characteristics such as the stromal cell density, cell atypia, and nuclear division number.^3)^ Ozello et al. reported that 1%–2% of PTs are complicated by breast cancer.^4)^

Here, we report the rare case of a 21-year-old female with breast cancer within a PT.

## CASE PRESENTATION

**Fig. 1:**
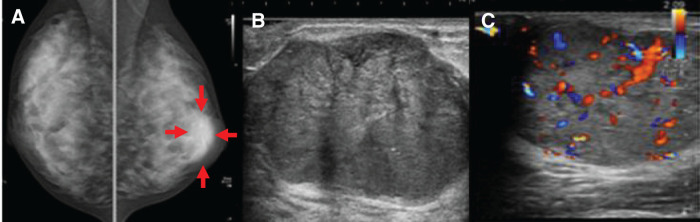
(**A**) Mammography: a mass just below the nipple in the left breast (arrows). (**B**) Ultrasonograpy: a lobular hypoechoic mass, 53 mm in size with multiple slit-like structures inside the mass. (**C**) Ultrasonography: blood flow inside the mass was rich.

**Fig. 2:**
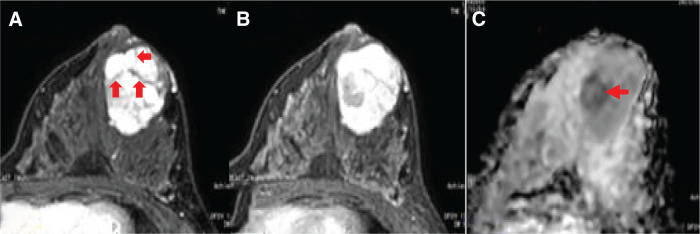
Dynamic MRI: (**A**) early phase. (**B**) Late phase. The tumor showed heterogeneous enhancement from the early phase, and contrast enhancement persisted in the late phase. Dark internal septa were observed inside (arrows). (**C**) Diffusion image: ADC map. Within the mass, a solid area of about 15 mm in diameter with diffusion-limited was observed (arrow).

**Fig. 3:**
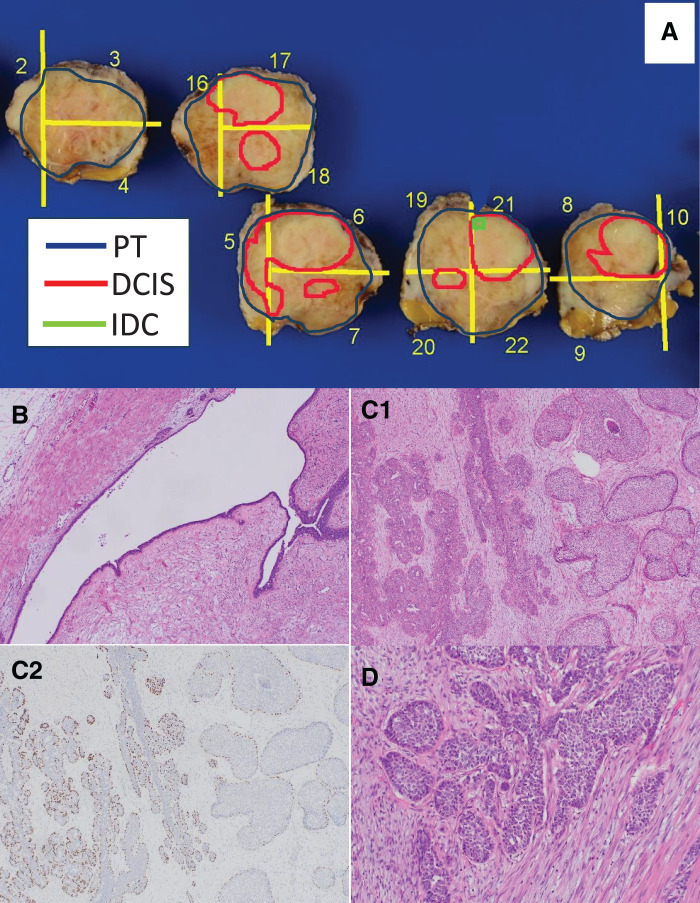
(**A**) Macroscopic of the resected specimen with mapping: a well-defined solid white mass measuring 47 × 35 × 40 mm. (**B**) Leaf-like structures typical of a phyllodes tumor (H-E×20). (**C**) The boundary between phyllodes tumor and DCIS: (C1) H-E×40, (C2) p63. The left side was the component of phyllodes tumor, while the right side was DCIS with comedo necrosis. (**D**) Invasive ductal carcinoma component (H-E×100). DCIS, ductal carcinoma in situ; IDC, invasive ductal carcinoma, PT, phyllodes tumor

The patient was a 21-year-old female with no significant medical history. Her grandmother had a history of breast cancer. She was diagnosed with a mass in her left breast and referred to the hospital. US revealed a 37 × 27 mm lobulated mass at the 3 o'clock position, which was considered an FA or PT and was under observation. Three months later, the mass had increased to 46 × 31 mm, and was referred to our hospital on suspicion of PT. When she visited our hospital, the mass was 40 × 40 mm, with good mobility and no palpable axillary lymph nodes. Mammography revealed a well-defined, high-density mass (**Fig. 1A**). US revealed a 53 × 41 × 35 mm lobulated, hypoechoic mass with multiple slit-like structures inside the mass and rich blood flow (**Fig. 1B** and **1C**). Dynamic MRI showed a mass with a heterogeneous high signal intensity on T2WI and persistent enhancement. In addition, dark internal septa, which are often observed in FAs, were observed (**Fig. 2A** and **2B**). However, a solid 15-mm diameter area with a heterogeneous low signal was observed in part of the mass, which was diffusion-limited (**Fig. 2C**). Therefore, we considered the possibility of a PT that was more than a borderline malignancy, rather than an FA. A core needle biopsy of the mass was performed. Pathologically, fibroepithelial lesions were identified, and FA and PT were considered differential diagnoses. FA was more strongly suspected because of the poor stromal changes. Based on the imaging and pathological results, we planned a tumorectomy with a safety margin. Pathological macroscopic examination revealed a well-defined solid white mass measuring 47 × 35 × 40 mm (**Fig. 3A**). Histologically, the mass appeared to be a mixture of epithelial and fibrotic stromal components. The mass was diagnosed as a benign PT, owing to the low cell density of the fibrostromal component, unremarkable cellular atypia and cell division, and scattered leaf-like structures (**Fig. 3B**). However, atypical cells with comedo necrosis proliferated in some areas (25 × 20 mm) (**Fig. 3C**). The atypical cells were positive for myoepithelial markers (p63), except for a portion of the area (approximately 2 mm), which was negative. Based on the above, the overall diagnosis of the mass was “IDC, with a predominance of intraductal carcinoma within a benign PT.” The cancer was encased by the PT, negative for resection margins, estrogen receptor-positive, progesterone receptor-positive, human epidermal growth factor receptor type2 score-negative, and Ki-67 score of 5%. The patient was followed up postoperatively without any additional treatment.

Considering her family history and her age at breast cancer diagnosis, we recommended her to undergo genetic counseling and *BRCA1/BRCA2* genetic testing, however, she refused to do either.

## DISCUSSION

We encountered a rare case of breast cancer in a PT in a patient aged 21 years. In the preoperative diagnosis, we considered the possibility of malignant PT based on the clinical course and imaging findings but did not consider the possibility of breast cancer within the PT. As the invasive cancer was very small and the resection margins were negative, she was treated without any postoperative treatment and did well, with no signs of recurrence.

A PubMed search from January 1990 to December 2023 using the keywords “phyllodes tumor,” “breast cancer,” “ductal carcinoma,” and “lobular carcinoma” yielded 52 reports. These reports involved cases ranging from 19 to 79 years old, with a mean age of 50.3 years. Among them, only 10 cases had an age at onset under 30 years. To investigate whether younger patients had specific characteristics, we summarized these 10 cases and our case in **Table 1**.^5–15)^ No obvious features were specific to younger patients, but 7 of the 10 patients were aware of rapid mass growth on a weekly-to-monthly basis. Four patients were diagnosed with cancer preoperatively and were found to have coexisting PT postoperatively. The other six patients were operated on following a diagnosis of PT or FA, and postoperative pathology revealed concomitant cancer. In three of the latter six cases, no additional resection was performed because of negative margins. In the remaining three cases, additional resection or SN was performed after the initial surgery, but no cancer remnants were found in the additional resected specimens. The reason for additional resection was the proximity of the breast tumor to the resection margin or the presence of invasive cancer within the mass, although the resection margin was negative. No cases were identified in which “concurrent PT and cancer” was predicted preoperatively. As a postoperative treatment, four of the six patients with IDC received systemic therapy, and one received only RT. Of the four DCIS patients, two received hormone therapy, and none received RT, except for one patient with unknown details. It was rare for cancer to coexist within a PT, and furthermore, our case presented at the young age of 21, which was exceptionally young for breast cancer. The cancer was confined within the PT, with no exposure, and was a predominantly intraductal component with a 2-mm invasion. Considering these factors, and after consultation with the patient, we decided on surveillance without additional treatment.^16)^

**Table 1:** Cases of cancer developing within phyllodes tumors in patients under 30 years of age

Report year	Author	Age	Complaint	Size (mm)	Preoperative diagnosis	Operation	PT category	Carcinoma	Size of carcinoma	Additional surgery	Radiotherapy	Adjuvant therapy
1997	Kurokawa	20	Awareness	60	PT	Tm	Border	IDC	Focal	NSM	–	–
2004	Parfit	26	Awareness	33	Carcinoma	Tm	Benign	DCIS and IDC	Unknown	Ax	+	CT + HT
2004	Senga	28	Awareness	48	PT	Tm	Benign	DCIS	Focal	–	–	–
2010	Inoue	26	Awareness	33	PT	Tm	Benign	IDC	Focal	–	–	–
2010	Kuo	24	Increase in follow-up	100	Fibroepithelial lesion	Tm	Border	IDC	Unknown	Bt + SN	–	CT + HT
2014	Colakoglu	19	Awareness	18	PT	Tm	Benign	DCIS	8 mm	–	+	HT
2016	Chopra	23	Awareness	50	PT	Tm	Benign	DCIS	Focal	Bp	Unknown	Unknown
2018	Lui	19	Awareness	51	DCIS	Bp+SN	Benign	DCIS	Unknown	–	–	HT
2021	Ilhan	28	Awareness	34	IDC	Bt+SN	Border	IDC	14 mm	–	–	HT
2021	Park	21	Awareness	24	IDC	Bp+SN	Border	IDC	Unknown	–	+	HT
2025	Present case	21	Awareness	47	Fibroepithelial lesion	Tm	Benign	IDC with predominant DCIS	DCIS 25 mm	–	–	–
									IDC 2 mm			

Ax, axillary dissection; Bp, breast-conserving surgery; Bt, mastectomy; CT, chemotherapy; DCIS, ductal carcinoma *in situ*; HT, hormonal therapy; IDC, invasive ductal carcinoma; NSM, nipple-sparing mastectomy; PT, phyllodes tumor; SN, sentinel lymph node biopsy; Tm, tumorectomy

As chemotherapy is generally considered almost completely ineffective against PTs, postoperative treatment of breast cancer within PT is determined by the status of the concomitant breast cancer.^7)^ Owing to the infrequency of breast cancer complications in PTs, there are no clear treatment guidelines.^15,17)^ The treatment plan should be based on the degree of cancer progression and biology, as in the case of breast cancer occurring in isolation. In case reports from Japan, many patients did not undergo additional resection, RT, or postoperative adjuvant therapy because the breast cancer is often very small.^7,18)^ Careful follow-up is necessary in such cases.

SN is often not performed, even when additional resection is performed. This is because depending on the location of the tumor, the lymphatic flow may change postoperatively owing to subcutaneous dissection, making accurate biopsy impossible in some cases.^19)^

According to reports from Japan, DCIS, IDC, and lobular carcinoma *in situ* are the most frequent histological types of breast cancer within PTs.^20)^ Very rare cases of squamous cell carcinoma, tubular carcinoma, and invasive lobular carcinoma within a PT have also been reported.^20)^ Although these multiple histological types may be combined within a PT, DCIS alone is the most common complication.^18,20)^ There are several theories regarding the coexistence of PTs and breast cancer. Recent molecular biology studies have reported that cells in both the epithelial and stromal components of PT may harbor mutations in genes that induce malignancy.^21)^ Therefore, genetic mutations within the hyperplastic epithelium of PT are considered a possible factor for ductal carcinoma. Other factors include accidental merger and the involvement of female hormones, because some PTs are hormone receptor-positive. However, no consensus has yet been reached.^5,18)^

Regarding image characteristics, contrast-enhanced MRI of the PT generally shows a markedly contrast-enhanced lobulated mass on T1WI and a slightly high-to-high signal on RT; however, the internal structure findings are inconsistent, depending on the amount of fibrous components in the stroma, and distinguishing PT from FA is difficult.^22)^ Malignant PT is generally considered highly suspicious with high T1WI signals, low-to-equal T2WI signals, low ADC, and prominent cystic structures.^23)^ In our case, considering the location and size of the carcinoma within the PT, the area that showed a low ADC on preoperative MRI may have coincided with the location of the carcinoma. A case was reported in which a carcinoma within a PT was suspected on preoperative imaging, and the same area was accurately diagnosed by needle biopsy; however, such reports are very rare.^24)^

Young breast cancer patients have a high probability of a genetic predisposition, and identifying hereditary diseases, such as HBOC with pathogenic variants in the *BRCA1*/*BRCA2* genes and Li-Fraumeni syndrome with pathogenic variants in the *TP53* gene.^25)^ In this case report, the patient’s family history included a maternal grandmother diagnosed with breast cancer at an advanced age, but there was no other HBOC-related family history. This means the patient does not meet the criteria for Chompret *TP53* screening, but is eligible for HBOC screening.^26,27)^ The proportion of cases diagnosed with HBOC in families with a history like this case was 18.6% according to a report by Japanese HBOC Consortium Registration Project.^27)^ We suggested genetic counseling and *BRCA1/BRCA2* genetic testing to her, however, she refused to do either. We will provide information about genetic testing at an appropriate time, as her perspective on genetic testing may change with her stage of life.

## CONCLUSIONS

Herein, we report the case of a 21-year-old female with breast cancer occurring within a PT. Predicting the presence of cancer preoperatively was difficult based on the patient’s young age and imaging findings.
